# Cytotoxic Effects of Mangosteen Pericarp Extracts on Oral Cancer and Cervical Cancer Cells

**DOI:** 10.31557/APJCP.2020.21.9.2577

**Published:** 2020-09

**Authors:** Sunitha Janardhanan, Jaideep Mahendra, Little Mahendra, Nalini Devarajan

**Affiliations:** 1 *Meenakshi Academy of Higher Education and Research, Meenakshi Ammal Dental College and Hospital, Chennai, India. *; 2 *Faculty of Dentistry, Meenakshi Academy of Higher Education and Research Meenakshi Ammal Dental College and Hospital Chennai Chennai, India. *; 3 *Maktoum Bin Hamdan Dental University College, Dubai, United Arab Emirates. *

**Keywords:** Mangosteen, cervical cancer, cytotoxic effects

## Abstract

**Background:**

Despite immense advancements in treatment modalities, cancer remains a dreadful disease until the present. The major influencing factors behind the increased mortality rate of cancer are increased drug resistance and severe adverse effects caused by conventional cancer therapies. To overcome these limitations, the current medical field is focusing more on natural phyto-derived molecules to mitigate cancer. Mangosteen is a phytotherapeutic with potent anti-inflammatory and antioxidant properties. In the present study, we investigated the anticancer potential of the crude ethanolic extract of mangosteen against two dreadful forms of cancers, namely, oral cancer and cervical cancer, *in vitro*.

**Methodology:**

The pericarp of *Garcinia mangostana* or mangosteen was removed, air-dried, ground to fine powder, and macerated with ethanol. The extract obtained was then filtered and extracted with water for 48 h. The aqueous fraction thus obtained was then concentrated with a rotary evaporator at 40°C and dried with a freeze dryer. The anticancer efficacy of these extracts was investigated in human tongue squamous cell carcinoma (H357) cells and cervical cancer cells (HeLa) using the MTT assay, TUNEL assay, western blotting, and flow cytometry techniques.

**Results:**

The crude mangosteen pericarp extract (MPE) significantly inhibited the growth of H357 and HeLa cells in a dose-dependent manner. Moreover, mangosteen induced early apoptosis in these cells after 48 h of incubation. Mangosteen also upregulated the expression of pro-apoptotic proteins, including caspases and Bax, and downregulated the expression of anti-apoptotic protein Bcl-2.

**Conclusion:**

The MPE exerted significant cytotoxicity against the H357 and HeLa cells in a dose-dependent manner and promoted their apoptosis. Hence, this natural phytoextract can be considered a potent anticancer agent for treating oral cancer and cervical cancer.

## Introduction

Oral cancer is widespread in the Indian subcontinent, with India contributing 7.8% to the global cancer burden and 8.3% of global cancer deaths (Prasad, 2014). Oral cancer is the sixth most common cancer in the world (Singh et al., 2017) and the second most common cancer in men after lung cancer (Gupta and Johnson, 2017). The majority of oral cancers (75%) are linked to excessive use of tobacco and alcohol consumption. 

Cervical cancer in women continues to be the leading cause of death worldwide, despite efforts for the early detection of cervical cancer (Kaarthigeyan, 2012). It is the fifth most common cancer in the world. In India, cervical cancer is the second most common cancer and accounts for 1/4 of all cervical cancer deaths (Torre et al., 2017). Oral cancer and cervical cancer thus continue to cause increased levels of mortality and contribute to the economic burden in the Indian subcontinent. 

Cancer still remains a disease that is difficult to treat, with only a 20% cure rate after treatment. India contributes to about 1/3 of global cancer deaths. The conventional modes of treatments include surgery, radiation, and chemotherapy (Ramamoorthy et al., 2015). These treatment modalities induce a significant amount of morbidity in treated individuals with the additional drawback of frequent recurrences. In addition, these treatments are too expensive and cause severe adverse effects, thus lowering the quality of life after treatment (Vyas et al., 2010). There is hence a dire need to explore novel herbally derived molecules that can act as supplements in treating oral cancer and thus can overcome the drawbacks. 

*Garcinia mangostana*, a fruit native to South East Asia, is used in classical ayurvedic formulations. The pericarp of the fruit is recommended for the treatments of diarrhea, dysentery, and skin infections. It is also used as an astringent in the treatment of gum diseases (Janardhanan et al., 2017). The fruit rind or pericarp of mangosteen possesses anticarcinogenic property based on *in vitro* studies on liver, breast, colorectal, lungs, cervical, and, prostate cancer cell lines (Orozc et al., 2013; Supason et al., 2014). Mangosteen exerts its anticancer potential by promoting cell cycle arrest, inhibiting cell proliferation, inducing apoptosis, and abrogating tumour cell adhesion, migration, and metastasis (Chaverrie et al., 2008). 

The pericarp of mangosteen is rich in bioactive agents, such as xanthones, flavonoids, procyanidin, and benzophenones, which are said to contribute to its anti-inflammatory, antibacterial, and anticancer effects (Aizat et al., 2018). The percentage of xanthones is high in the pericarp rather than the pulp of the fruit (Sukatta et al., 2013). Among the xanthones, alpha-mangostin, beta-mangostin, gamma-mangostin, mangostinone A, mangostinone B, and mangostenol are present abundantly in the pericarp. These xanthones are responsible for the anticancer action of mangosteen on leukaemia, prostrate, breast, and colon cancer cell lines (Jindarat, 2014).

Although the chemoprotective potential of the mangosteen pericarp extract (MPE) has been widely studied in different types of cancer, the cytotoxic effects of this herbal medicine against the most dreadful forms of cancers, including oral cancer and cervical cancer, are not yet known. Hence, the present study explored the role of MPE on the human oral squamous cell carcinoma cell line of the tongue (H357) and cervical cancer cell line (HeLa) *in vitro*. The pro-apoptotic potential of MPE on the HeLa and H357 cell lines was evaluated by TUNEL (Terminal deoxynucleotidyl transferase dUTP Nick End Labelling) assay, annexin / propidium iodide (PI) flow cytometry, and western blot analysis of proteins involved in the mitochondrial apoptotic pathway. 

## Materials and Methods

*Preparation of MPE*

The study protocol was submitted to the Institutional Review Board and Institutional Ethical Clearance of MAHER University, and approval for the study (MAHER-MU-004-IEC/2016) was obtained. *G. mangostana *or mangosteen was botanically authenticated by a pharmacognosist from Siddha Central Research Institute (G17120701M). The pericarp of the fruit was removed, air-dried, and ground to a fine powder in a lathe. Then, 100 g of the pericarp powder was macerated with ethanol and allowed to stand for two days. The ethanolic extract was then filtered, and the dried residue was extracted with distilled water for 48 h and stored in a dark closed glass container. The obtained aqueous fraction was then concentrated with a rotary evaporator at 40°C and dried with a freeze dryer. The dried fraction was weighed and stored in Teflon-capped tubes at -20°C.

*Chemicals and reagents*

The chemical and reagents used for this study were of molecular biology grade and were procured from HiMedia and Sigma Aldrich, USA. Dulbecco’s modified eagle medium (DMEM, category number. L111), fetal bovine serum (FBS, category number RM10432), streptomycin and penicillin, and phosphate-buffered saline (D-PBSc, category number TL1006) were procured from HiMedia, France. PI (BD Biosciences, category number 556463), APO-DIRECT™ Kit (BD Biosciences, category number. 556381), and FITC Annexin V Apoptosis Detection Kit I (BD Biosciences, category number 556547), were procured from BD Biosciences, India.

*Phytochemical analysis*

The mangosteen pericarp samples were analysed to detect the presence of bioactive compounds, such as tannins, saponins, flavonoids, alkaloids, proteins, steroids, quinones, terpenoids, and cardiac glycosides.

*Cell culture*

H357 (human oral squamous cell carcinoma of the tongue) and HeLa (cervical cancer cell) lines were obtained from the American Type Culture Collection (Manassas, VA, USA) and maintained in DMEM supplemented with 10% FBS, 100 µg streptomycin, and 100 µg penicillin at 37°C in a humidified incubator under 5% of CO_2_ until they reached 80% confluence. 

*MTT assay*

The effect of mangosteen on the viability of H357 and HeLa cells was assessed by 3-4,5- dimethylthiazol-2-yl)-2,5-diphenyl tetrazolium bromide assay (MTT assay; #4060 HiMedia). Briefly, 1 × 10^6^ cells were seeded into a 96-well plate and allowed to grow for 48 h. Then, the cells were treated with varying concentrations of the MPE (0.78, 1.56, 3.12, 6.25, 12.5, 25, 50, 100, 200, and 250 μl) and incubated for 24 h at 37°C in a 5% CO_2_ atmosphere. After the incubation period, the effect of the MPE on cell viability was assessed as per cent cell viability and compared with vehicle-treated control cells, which were arbitrarily assigned 100% viability. The lowest concentration required to cause 50% cell growth inhibition (IC50) was determined via an interpolation from the dose response curve. The IC_50_ was employed as an optimal dose for studying the anticancer properties of MPE in HeLa and H357 cell lines. This experiment was performed in triplets, and the average values were estimated.

*Detection of DNA fragmentation by TUNEL assay*

TUNEL assay identifies DNA breaks by labelling the 3’-OH terminal of DNA with modified nucleotides to TdT or terminal nucleotidyltransferase. Briefly, 1 × 10^6^ cells were treated with the MPE for 48 h. After the incubation period, the supernatant and adherent cells were collected in a centrifuge tube and washed twice with 1× PBS. The cells were fixed with 70% ice-cold ethanol by incubating at -20°C for 30 min. The cells were resuspended in 1 ml of wash buffer and washed twice via centrifugation. 50 μl of the DNA labelling solution was added, and the cells were incubated at 37°C for 60 min. The cells were rinsed with 1.0 ml of the rinse buffer and centrifuged, and the supernatant was removed. The cell pellet was resuspended in 0.5 ml of a PI/RNase staining buffer and incubated for 30 min at room temperature. Controls were included in the study; for the negative control, TdT was omitted. Flow cytometry was performed to detect the DNA fragmentation.

Flow cytometry: HeLa and H357 cells (1 × 10^6^ cells/well) were seeded in a 6-well plate and treated with MPE for 48 h. After the incubation period, the cells were harvested, washed twice with PBS, gently resuspended in 100 μL annexin V-FITC binding buffer (1×), and incubated with 5 μL annexin V-FITC in dark for 10 min at 25°C. The cells were then centrifuged at 2,000 rpm for 5 min and gently resuspended in 500 μL annexin V-FITC binding buffer (1×), and 5 μL PI was added in an ice bath. The tagged cells were immediately analysed via flow cytometry through the CellQuest software (BD Biosciences). 

Western blot analysis: The H357 and HeLa cells were treated with 6.25, 12.5, and 25 μL MPE for 48 h and were lysed with lysis buffer for protein isolation. Protein estimation was performed using the Bradford protein assay method. 20 µg of protein was then run in 4% SDS-PAGE and transferred to 0.4 µm PVDF membrane via the gel sandwich technique under a bio-ice cooling unit. The blots were then incubated for 1 h in a blocking buffer and then incubated with antibodies (anti-caspase-3, anti-caspase-6, anti-bcl-2, and anti-bax-2) at 4°C. The membranes were then incubated for 1 h with respective secondary antibodies at room temperature. Finally, the membranes were incubated with chromomeric substrate 3,3′-diaminobenzidine (DAB), and the bands were visible within 3–5 minutes of incubation.

*Statistical analysis*

The Kolmogorov–Smirnov and Shapiro–Wilks normality test results reveal that all variables do not follow a normal distribution. Therefore, to analyse the data, non-parametric methods were applied. To compare values between the H357 and HeLa groups, the Mann-Whitney test was performed. To analyse the data, SPSS (IBM SPSS Statistics for Windows, version 25.0, Armonk, NY: IBM Corp., released 2017) was used. The significance level was fixed as 5% (α = 0.05). The Mann–Whitney test for MTT showed that the MPE significantly inhibited the H357 and HeLa cells at varying concentrations of 0.78, 1.56, 3.12, 6.25, 12.5, 25, 50, 100, 200, and 250 μg. The results of the TUNEL assay were also statistically significant for the MPE-treated H357 and HeLa cell lines. In the apoptotic assay using annexin V and PI, the results were statistically significant only in live cells, early apoptosis, and necrotic cells in the H357 and HeLa cell lines.

## Results

Phytochemical analysis: Phytochemical analysis showed that the MPE is rich in tannins, flavonoids, proteins, steroids, and cardiac glycosides (Table 1).

**Figure 1:**
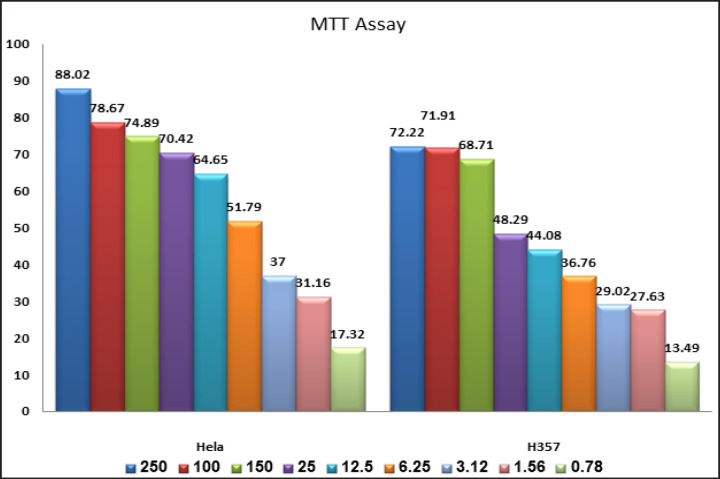
Graphical Representation of the MTT Assay Results Showing the Cytotoxic Effect of Different Concentrations of Mangosteen Pericarp Extract on HeLa and H357 Cell Lines

MTT assay: The cytotoxicity effects of the MPE on H357 and HeLa cells were investigated by MTT assay. MPE reduced the viability of H357 cells in a dose-dependent manner (0.78, 1.56, 3.12, 6.25, 12.5, 25, 50, 100, 200, and 250 µg/ml), and the IC_50_ value was found to be 9.8 µg/ml. Hence, 9.8 µg was used for further investigation. Similarly, in HeLa cells, mangosteen exhibited an IC50 value of 7.5 µg/ml. Hence, 7.5 µg was used for further studies on HeLa cells (Table 2 and Figure 1).

*Detection of DNA fragmentation by TUNEL assay*

TUNEL assay measures the DNA strand breakdown and provides information on cell apoptosis. Table 3 shows the chemoprotective effect of mangosteen on H357 and HeLa cells. This potent drug exhibited an apoptotic rate of 20% in the H357 cells (p < 0.0495) and 60% in the HeLa cells (p < 0.0495) (Figure 2).

Flow cytometry analysis: Annexin V-FITC and PI flow cytometry assay were conducted to evaluate the pro-apoptotic potential of MPE. Here, the exposure of the phospholipid layer of the cell membrane in H357 and HeLa cancer cells after treatment with MPE were visualized by its binding to annexin V. Annexin V/PI dual staining showed that the MPE could trigger early apoptosis, late apoptosis, and necrosis in HeLa cells (Table 4). However, in H357 cells, the MPE induced early and late apoptosis without any necrosis. Figure 3 indicates that the number of cells undergoing apoptosis in the early stage was significantly high in the MPE-treated H357 and HeLa cells when compared with their control cells. 41.84% of the HeLa cells showed cell death when treated with the MPE. Among these cells, 35.60% of the cells underwent early apoptosis and 0.5% necrosis. In H357 cell lines, 52.46% of the cells showed apoptosis. Of these, 46.37% underwent early apoptosis and 0.41% of the cells underwent necrosis. 

*Western blotting*

Western blot analysis was performed to study the effect of mangosteen pericarp on the expression of caspase 3, caspase 9, Bcl-2, and Bax proteins in H357, and HeLa cells (Figure 4). There was a dose-dependent increase in the expression of pro-apoptotic proteins, including caspase 3, caspase 9, and Bax, and reduction in antiapoptotic proteins, such as Bcl-2, in the MPE-treated HeLa and H357 cells at concentrations of 6.25 µg, 12.5 µg to 25µg/ml . 

**Table 1:** Phytochemicals Present in the Crude Ethanolic Extract of Mangosteen Pericarp

S.No	Contents	Sample
1	Tanins	+
2	Saponin	-
3	Flavonoids	+
4	Alkaloids	+/-
5	Proteins	+
6	Steroid	+
7	Quinones	-
8	Terpenoids	-
9	Cardio glycosides	+

**Figure 2:**
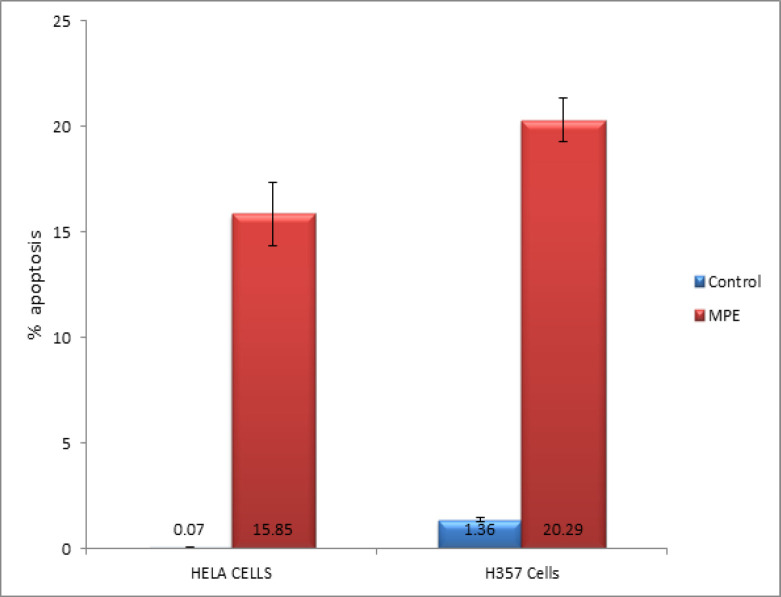
Graphical Representation of the Pro-Apoptotic potential of MPE on H357 and HeLa cells

**Figure 2 A:**
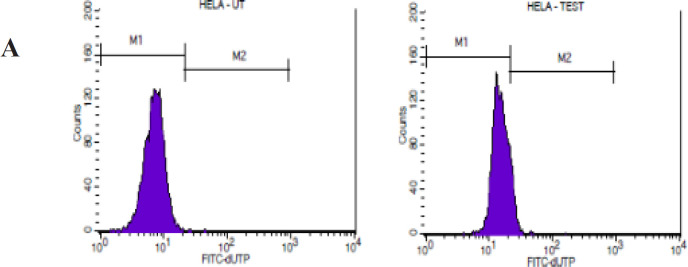
Effect of MPE on HeLa Cells as Shown in TUNEL Assay

**Figure 2 B:**
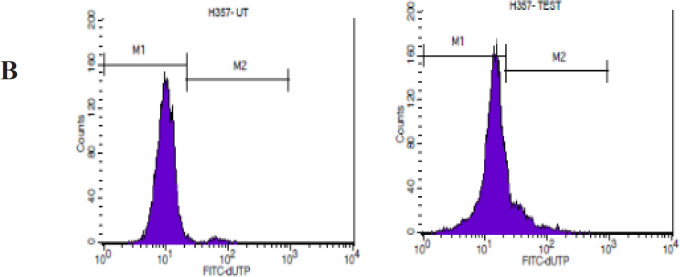
Effect of MPE on H357 Cells as Shown in TUNEL Assay

**Table 2:** Cell Viability Assay in HeLa Cells: MPE Dose Dependently Reduced the Viability of Both HeLa and H357 Cells

MPE Concentration (μg/ml)	% of cell death in HeLa cells	% of cell death in H357 cells
250	88.02+1.53	72.22+0.73
100	78.67+1.01	71.91+1.27
50	74.89+1.06	68.71+0.73
25	70.42+1.77	48.29+1.54
12.5	64.65+0.61	44.08+0.85
6.25	51.79+1.53	36.76+0.80
3.125	37.00+1.06	29.02+1.93
1.562	31.16+0.64	27.63+1.97
0.781	17.32+0.79	13.49+3.83

**Figure 3:**
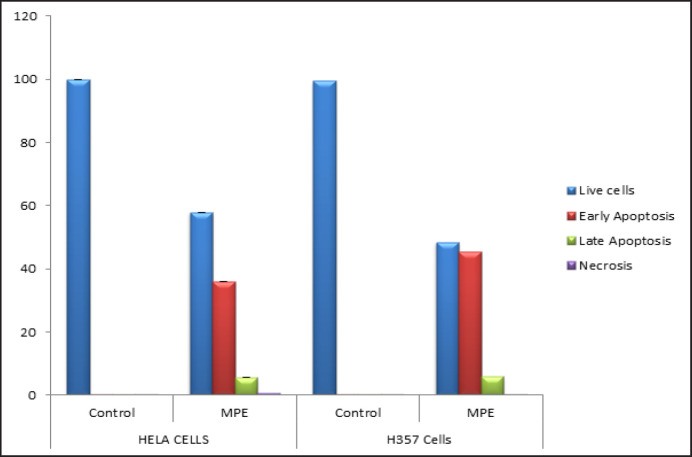
Flow Cytometry Analysis of the Pro-Apoptotic Potential of Mangosteen on HeLa and H357 Cells

**Figure 3A and 3B:**
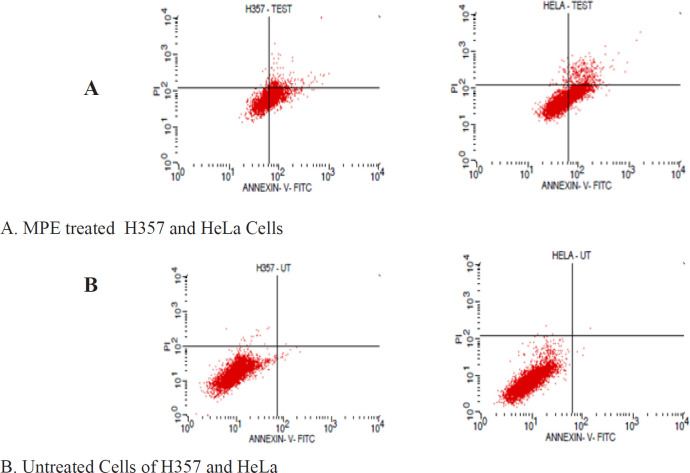
MPE Induces Significant Amount of Early Apoptosis in Both HeLa and H357 Cells.

**Table 3:** Tunnel Assay of the Pro-Apoptotic Potential of MPE on HeLa and H357 Ccells

Cell Lines	Control group	MPE treated group	P value
HeLa cells	0.07+0.015	15.85+1.5	0.0495(S)
H357 cells	1.36+0.086	20.29+1.037	0.0495(S)

p<0.05- Significant (S)

**Figure 4A:**
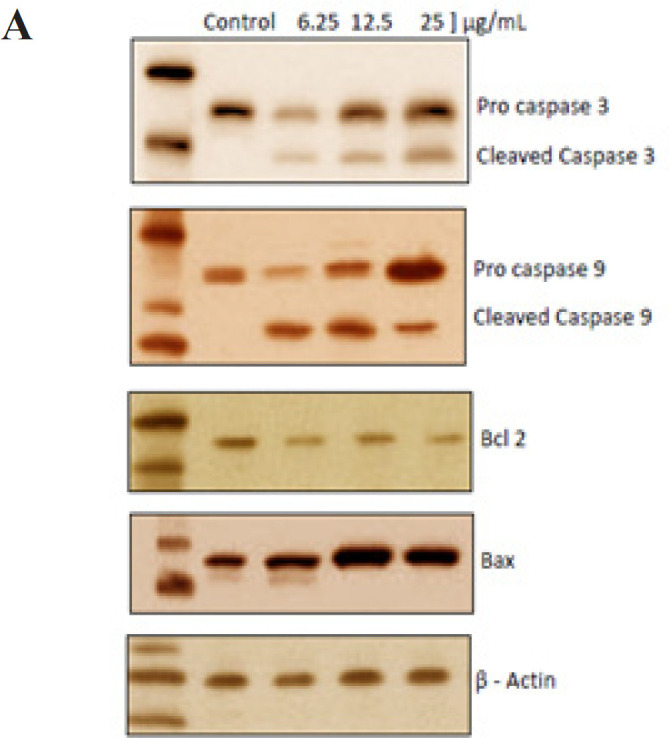
Effect of Mangosteen on the Expression of Proteins Involved in Apoptosis in HeLa Cells

**Table 4:** Flow Cytometry Analysis of the Pro-Aapoptotic Potential of MPE on HeLa and H357 Cells

Annexin V/ Pi Staining	Control Group	MPE Treated Group	*P*-value
Cell Lines			
Live Cells	99.88+0.02	57.85+0.566	0.495
Early Apoptosis	0.04+0.015	36.00+0.4	0.495
Late Apoptosis	0.02+0.015	5.47+0.321	0.495
HeLa Cells			
Necrosis	0.05+0.010	0.68+0.131	0.495
Live Cells	99.49+0.049	48.41+0.833	0.495
Early Apoptosis	0.23+0.058	45.32+1.0	0.046(S)
H357 Cells			
Late Apoptosis	.03+0.006	5.89+0.919	0.046(S)
Necrosis	0.25+0.114	0.37+0.154	0.275

P<0.05-Significant (S)

**Figure 4B:**
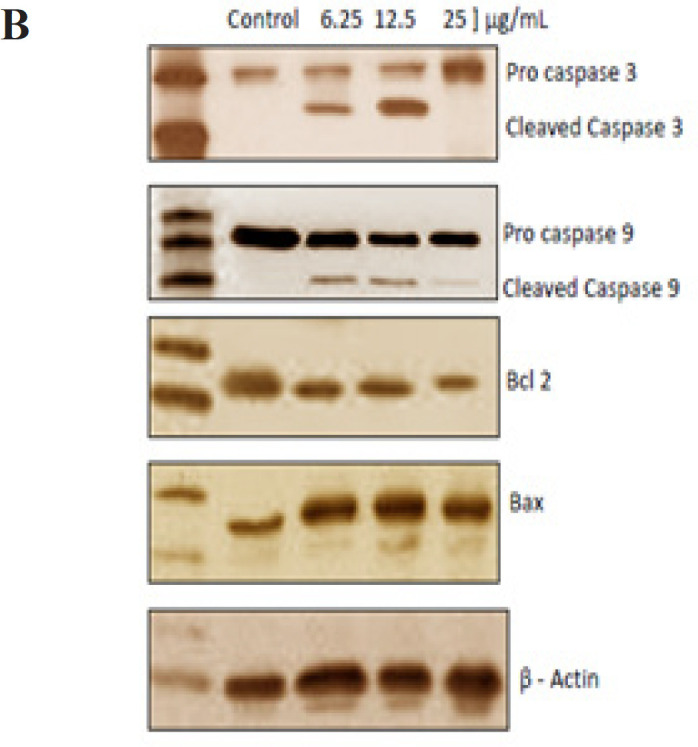
Effect of Mangosteen on the Expression of Proteins Involved in Apoptosis in H357 Cells

## Discussion

Despite the efficient mitigation of cancer cells by conventional cancer drugs, chemotherapy is always associated with inadvertent damage to adjacent normal cells (Ramamoorthy et al., 2015). Hence, the search for an ideal anticancer therapy that selectively kills the tumour cells without causing any adverse effect to the host cells still continues. Naturally obtained products are considered a boon for cancer treatment due to their effective action on cancer cells and minimal cytotoxicity to normal cells (Greenwell and Rahman, 2015; Rayanet al., 2017). In this regard, the mangosteen extract, which is rich in xanthones, such as alpha-mangostin, gamma-mangostin, mangostinone, and gartanin, has been proven to be an effective anticancer agent (Kwak et al., 2016). 

The therapeutic property of mangosteen is attributed to the presence of xanthones, a flavonoid present in the pericarp. Interestingly, our phytochemical analysis also shows that the MPE is rich in tannins, flavonoids, steroids, proteins, and cardiac glycosides. 

The study investigated the chemoprotective effects of ethanolic crude MPE on H357 and HeLa cells. Mangosteen has been found to severely affect the viability of these cells in a dose-dependent manner. The treatment with the MPE resulted in a steady decrease in the count of viable cells as the extract concentration was increased from 1.56 to 250 μg/mL. The findings of the study were in accordance with those in the studies performed by Kwak et al. wherein alpha- mangostin, a mangosteen derivative, altered the cell viability in human oral squamous cell carcinoma cell lines, namely, HSC2, HSC3, and HSC4, in a dose- and time-dependent manner.17

As observed in H357 oral cancer cells, the cytotoxicity of the ethanolic MPE on HeLa cell lines has also been shown to increase dose dependently from 1.56 to 250 μg/mL. The same results have also been obtained with alpha-mangostin and, the standard anticancer drug cisplatin on HeLa cell lines was evaluated. Cell lines were preincubated with alpha-mangostin for 24 h, followed by coincubation with cisplatin for 24 h. In both cases, the cell viability showed a cytotoxic effect in a concentration-dependent manner. In addition, the alpha-mangostin was protected from cytotoxicity at an extremely low concentration of 5–15 μM (Rojas et al., 2016). In another study, mangosteen extract in the form of GME-MC (*G. mangostana* with cellulose) and GME-EC/MC (*G. mangostana* with ethyl and methyl cellulose) polymeric nanoparticles on HeLa cell lines was studied. This finding was also confirmed to be dose dependent. As an added advantage, introducing mangosteen as a nanoparticle also improved the bioavailability of mangosteen and increased its cellular uptake (Supason et al., 2014). Compared with the effect of the MPE on the H357 and HeLa cell lines, the MPE at concentrations between 1.56 and 250 μg/mL was significantly more cytotoxic to the HeLa cell line than to the H357 cell line.

The TUNEL assay results showed that the MPE can trigger apoptosis in H357 and HeLa cell lines (p < 0.0495). To further monitor the progression of apoptosis and identify the cells in various stages of apoptosis, annexin V/PI assay was performed. Our results show a significant increase in cells showing early apoptosis in H357 and HeLa cells when compared with the control cells. This finding, in turn, provides direct evidence that MPE can induce early apoptosis, which can be attributed to the increase in membrane permeability and mitochondrial dysfunction. 

The regulation of apoptosis is performed by complex interactions between various proteins. Some of these proteins, such as Bax, promote apoptosis, and others, such as Bcl-2, are antiapoptotic in function. Caspases are key enzymes controlling apoptosis. Caspase 3 has a critical role in the apoptotic process and is activated by caspase 9. The expressions of Bax, Bcl-2, caspase 3, and caspase 9 were detected by the western blot analysis. The MPE induced an increased level of expression of pro-apoptotic protein Bax and decreased level of expression of antiapoptotic protein Bcl-2 in a concentration-dependent manner in both cell lines. The levels of caspase 3 and 9 also increased with an increase in the concentration of the MPE from 6.25 and 12.5 µg to 25 µg/ml. In a previous study, it was inferred that alpha-mangostin induces cell death through the generation of ROS, which activates the ASK/p38 signaling pathway leading to the rupture of the mitochondria, increased release of Bax and cytochrome C, and decreased level of Bcl-2. This causes the activation of the caspase-9/caspase-3 cascade, causing apoptosis of cervical cancer cells (Lee et al., 2017).

Although various studies have evaluated the anticancer effects of mangosteen on HeLa cell lines, this is the first study to investigate the chemotherapeutic potential of the MPE on H357 oral cancer cells. Our study has shown that crude alcoholic MPE is effectively cytotoxic to H357 cell and HeLa cell lines. The MPE causes DNA fragmentation in both cell lines, indicating the intrinsic pathway of apoptosis to be involved in cell death. In this study, an increase in early apoptosis can be attributed to membrane permeability and mitochondrial dysfunction. Furthermore, an increased level of Bax, caspase 3, and caspase 9 with a decreased level of Bcl-2 confirm the involvement of the intrinsic and extrinsic pathways of apoptosis. 

In conclusion, the present study demonstrates that ethanolic crude MPE is cytotoxic and can induce apoptosis on oral and cervical cancer cells. These results prove that mangosteen pericarp can act as a potential therapeutic anticancer agent. However, further research into the molecular mechanism involved and on in vivo models will provide substantial evidence to the anticancer potential of MPE.
